# The m^6^A Modification in Neurodegenerative Disease: A Cellular Perspective

**DOI:** 10.3390/cells14221820

**Published:** 2025-11-20

**Authors:** Shuowei Wang, Ziming Feng, Hongjin Wu, Shen Wang, Suping Qin, Xiaotian Wang, Feng Zhou, Kuiyang Zheng, Xufeng Huang, Xiaomei Liu

**Affiliations:** 1 Jiangsu Key Laboratory of Immunity and Metabolism, Jiangsu International Laboratory of Immunity and Metabolism, School of Basic Medical Sciences, Xuzhou Medical University, Xuzhou 221004, China; 2 First Clinical Medical College, Xuzhou Medical University, Xuzhou 221004, China; 3 School of Anesthesia, Xuzhou Medical University, Xuzhou 221004, China; 4 Department of Pathogen Biology and Immunology, School of Basic Medical Sciences, Xuzhou Medical University, Xuzhou 221004, China; 5 School of Medical, Indigenous, and Health Sciences, University of Wollongong, Wollongong, NSW 2522, Australia

**Keywords:** m^6^A, glial cells, neuron, neurodegenerative diseases

## Abstract

**Highlights:**

**What are the main findings?**

- m^6^A modification exerts cell-type-specific regulatory roles in central nervous system.
- Dysregulation of m^6^A modification is closely involved in the pathological progression of neurodegenerative diseases.

**What is the implication of the main finding?**

- The cell-type-specific molecular mechanisms of m^6^A provide novel potential therapeutic targets for neurodegenerative diseases.

**Abstract:**

N6-methyladenosine (m^6^A) is the most abundant internal RNA modification in eukaryotes and plays a critical role in gene expression regulation by influencing RNA stability, splicing, nuclear export, and translation. Emerging evidence suggests that dysregulation of m^6^A contributes to neuroinflammation, neurotoxicity, and synaptic dysfunction—key features of neurodegenerative diseases. This review aims to examine the role of m6A modification in neurodegenerative diseases from a cell-type-specific perspective. We systematically reviewed recent studies investigating m^6^A modifications in neurons and glial cells. Data from transcriptomic, epitranscriptomic, and functional studies were analyzed to understand how m^6^A dynamics influence disease-related processes. Findings indicate that m^6^A modifications regulate neuroinflammation and immune responses in microglia, modulate astrocytic support functions, affect myelination through oligodendrocytes, and alter m^6^A patterns in neurons, impacting synaptic plasticity, stress responses, and neuronal survival. These cell-type-specific roles of m^6^A contribute to the progression of neurodegenerative diseases such as Alzheimer’s disease (AD), Parkinson’s disease (PD), and Amyotrophic lateral sclerosis (ALS). Understanding m^6^A-modulated mechanisms in specific neural cell types may facilitate the development of targeted interventions for neurodegenerative diseases.

## 1. Introduction

N6-methyladenosine (m^6^A), the most common chemical epigenetic modification among mRNA post-transcriptional modifications, is a dynamic and reversible methylation modification that occurs at the N6 position of adenine, including methylation, demethylation, and recognition [1,2]. Regulators of m^6^A are primarily classified into three categories, including writers, erasers, and readers, which are responsible for methylation, demethylation, and recognition, respectively, and collaborate to dynamically control m^6^A modification and its biological functions. Writers, including methyltransferase-like enzyme 3 (METTL3), methyltransferase-like enzyme 14 (METTL14), WT1-associated protein (WTAP), and Vir-like m^6^A methyltransferase-associated protein (VIRMA), mediate the process of methylation, which can be reversed with erasers such as fat mass and obesity-associated protein (FTO) and AlkB homolog H5 (ALKBH5). Moreover, the recognition of m^6^A is mediated by readers, notably YTH structural domain family protein 1/2/3 (YTHDF1/2/3), Insulin-like growth factor-binding proteins (IGFBPs), and Fragile X Mental Retardation Protein 1 (FMR1) [3,4,5]. m^6^A modification, which is mainly enriched in the 5′untranslated region (5′UTR) and 3′untranslated region (3′UTR) [6], influences RNA splicing and mRNA stability [7,8]. Additionally, m^6^A can promote the transport of mRNA from the nucleus to the cytoplasm, enhance translation efficiency, and increase protein expression [9,10]. However, dysregulation of m^6^A modification, including aberrant activity of writers, erasers, and readers, plays a significant role in the pathogenesis of diseases, such as vascular diseases, malignant tumors, and neurodegenerative diseases [1,11,12,13].

**Figure 1:**
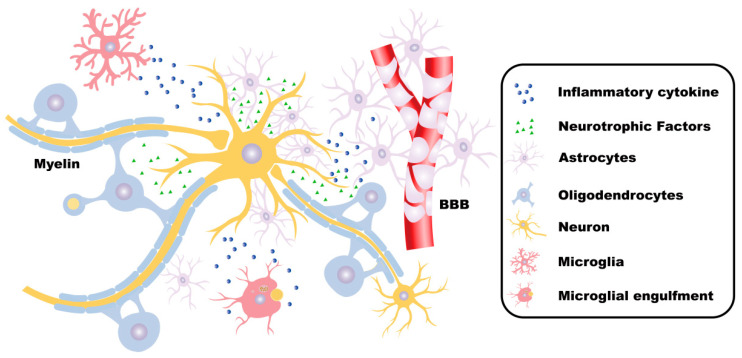
The functions of neurons and glial cells in the CNS. In the central nervous system (CNS), neurons receive, integrate, and transmit information. Astrocytes fulfill critical roles in maintaining CNS homeostasis, supporting neuronal survival, regulating synaptic transmission, forming the blood–brain barrier (BBB), and facilitating tissue repair processes. Microglia sense environmental changes and maintain physiological homeostasis. Under pathological conditions, microglia also secrete inflammatory mediators, phagocytize foreign substances, and participate in immune defense. Oligodendrocytes form myelin sheaths, which insulate and protect the axons of neurons. Moreover, oligodendrocytes provide nutritional support and metabolic maintenance for the axons, contributing to maintaining the integrity and normal function of the axons. These cells collaborate with each other to jointly maintain the normal structure and function of the CNS.

Neurodegenerative diseases, including Alzheimer’s disease (AD), Parkinson’s disease (PD), Huntington’s disease (HD), Multiple Sclerosis (MS), and Amyotrophic lateral sclerosis (ALS), which are characterized by neuroinflammation, progressive neurological dysfunction, and neuronal loss in the nervous system, result in cognitive decline and increased disability. Increasing studies have elucidated, m^6^A affects aging and brain cell development in AD [14]. Enhanced m^6^A methylation in the 5′UTR of Acyl-CoA synthetase long-chain family member 4 (ACSL4) has been shown to upregulate ACSL4 protein expression, thereby accelerating ferroptosis in dopaminergic neurons, which exacerbates both the onset and progression of PD [15]. It should be noted that resident cells in the central nervous system (CNS), including microglia, astrocytes, oligodendrocytes, and neurons, play different roles in the CNS and are involved in the development of neurodegenerative diseases (Figure 1).

Here, we summarize recent advances in understanding the dynamic regulation of m^6^A modifications within CNS-resident cells and their pivotal roles in inflammation and neurodegenerative diseases, highlighting the molecular mechanisms driving pathogenesis and the challenges impeding the development of targeted therapeutic strategies.

## 2. Microglia

### 2.1. Roles of Microglia in CNS

Microglia, the intrinsic immune cells of the CNS, are distributed extensively throughout the brain. The primary function of microglia is to sense environmental changes, sustain physiological homeostasis, and defend against harmful agents [16,17].

In the resting state, unstimulated microglia (M0 phenotype) contribute significantly to neuronal development and maintenance. Upon activation, microglia are differentiated into two distinct functional phenotypes: pro-inflammatory M1-like (M1) and anti-inflammatory M2-like (M2) [18,19]. M1 microglia produce inflammatory cytokines and drive neuroinflammation, which is implicated in the progression of neurodegenerative diseases [20]. In contrast, M2 microglia generally facilitate tissue repair and resolution of inflammation [21].

### 2.2. m^6^A Modifications in Microglia

Recent studies have identified that m^6^A RNA modifications undergo dynamic regulation across M0, M1, and M2 microglial phenotypes during neuroinflammation processes, along with numerous transcripts exhibiting significant upregulation or downregulation in m^6^A levels [19]. Notably, the m^6^A writer, METTL3, enhances the expression of the basic leucine zipper transcriptional factor ATF-like (BATF) via an IGF2BP2-dependent pathway in microglia-mediated neuroinflammation [22]. These observations highlight the potential importance of m^6^A modifications in regulating microglial function and suggest their involvement in the pathogenesis of neurodegenerative diseases.

### 2.3. m^6^A Modifications of Microglia in AD

AD, the most prevalent neurodegenerative disorder, is characterized by cognitive impairments, including deficits in memory and language expression. Its hallmark pathological features include intracellular neurofibrillary tangles (NFTs) due to abnormal tau protein aggregation and extracellular amyloid plaques formed by amyloid-beta (Aβ) protein accumulation [23,24,25,26].

Recent studies demonstrate significant alterations of m^6^A RNA methylation regulators in cortical tissues of AD patients. Compared to healthy controls, AD brains show substantial downregulation of m^6^A writers METTL3 (66.7%), METTL14 (74.0%), and WTAP (76.0%), as well as the eraser FTO (60%) and reader YTHDF1 (73.5%). These changes correlate with reduced m^6^A modifications in large pyramidal neurons and increased levels in GFAP-positive astrocytes and Iba-1-positive microglia [27]. Long-term circadian rhythm disruption regulates Hif3α m^6^A methylation at site 3632 and accelerates the progression of AD through the Hif3α/Lysine demethylase 3A (KDM3A)/TGF-β1 axis [28]. Furthermore, downregulation of FTO activates the Notch1–HES1 pathway, thereby triggering an immune response and promoting the progression of AD [29].

**Figure 2:**
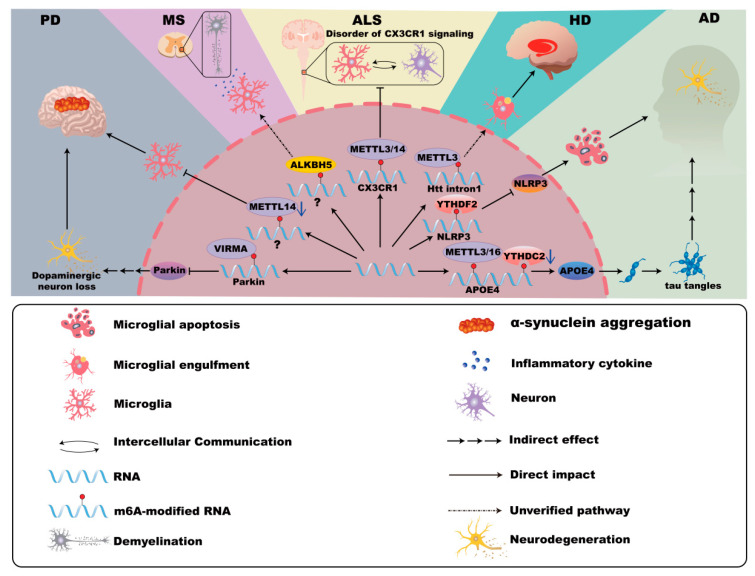
m^6^A modifications in microglia in neurodegenerative disease. In Alzheimer’s disease (AD), the expression of APOE4 is associated with YTHDC2, METTL3, and METTL16, promoting neurodegeneration. Furthermore, YTHDF2 inhibits microglial NLRP3/caspase-1/GSDMD signaling, potentially mitigating the damage associated with AD in the central nervous system. In Parkinson’s disease (PD), VIRMA-mediated m^6^A methylation suppresses Parkin translation, enhancing microglial activation, dopaminergic neuron loss, and neuroinflammation. Mettl14 deletion in the substantia nigra intensifies microglial activation and exacerbates PD pathology. In Huntington’s disease (HD), the abundant m^6^A modifications in mHTT transcripts may be related to the pathogenic mechanism of microglia. In Multiple Sclerosis (MS), the m^6^A modification mediated by ALKBH5 may have the possibility of influencing the microglial pyroptosis and inflammation. In Amyotrophic lateral sclerosis (ALS), m^6^A modifications in CX3CR1 of microglia disrupt the communication between neurons and glial cells.

The genetic risk factor apolipoprotein E (APOE4), abundantly expressed in microglia, contributes significantly to AD pathogenesis. APOE4 expression in microglia correlates with the downregulation of YTHDC2 and the upregulation of METTL3 and METTL16, facilitating tau-associated neurodegeneration independently of triggering receptor expressed on myeloid cells 2 (TREM2). Notably, conditional deletion of APOE4 from microglia ameliorates plaque pathology [30,31,32,33], indicating a crucial role for m^6^A regulators in modulating APOE4 expression and AD progression. Moreover, m^6^A-YTHDF2 inhibits microglial NLRP3/caspase-1/GSDMD signaling, potentially mitigating AD-related CNS damage [34]. In microglia, the METTL3/IGF2BP2/IκBα axis modulates microglia M1/M2 polarization, thus contributing to inflammation and neuronal damage in AD [35]. Furthermore, METTL3-mediated activation of the TRAF6/NF-κB pathway via m^6^A promotes neuroinflammation in microglia, highlighting a novel target for therapeutic strategies in AD [36,37,38,39] (Figure 2).

### 2.4. m^6^A Modifications of Microglia in PD

PD, the second-most common neurodegenerative disorder, is characterized by dopaminergic neuron loss and pathological α-synuclein aggregation, resulting in motor dysfunctions such as tremors, bradykinesia, rigidity, and gait disturbances [40,41,42,43,44,45,46]. Studies show that reduced m^6^A modification levels and decreased expressions of METTL3, METTL14 and YTHDF2 in peripheral blood mononuclear cells (PBMCs) from PD patients emphasize m^6^A dysregulation’s role in PD pathogenesis [47].

Microglial activation contributes significantly to α-synuclein pathology and its propagation. For instance, VIRMA-mediated m^6^A methylation suppresses Parkin translation, thereby enhancing microglial activation and neuroinflammation [3,48,49]. Furthermore, Mettl14 deletion in the substantia nigra intensifies microglial activation and exacerbates PD pathology [50,51]. These findings underscore the involvement of m^6^A modification in microglia-driven neuroinflammation in PD (Figure 2).

### 2.5. m^6^A Modifications of Microglia in HD

HD, caused by an expanded CAG repeat within the *Htt* gene, leads to mutant huntingtin (mHTT) protein formation, neuronal toxicity, and widespread neurodegeneration, notably affecting the functions of GABAergic medium spiny neurons (MSNs) and white matter [52,53,54,55,56]. Studies from Hippocamps of HD mice reveal hypermethylation of m^6^A in synaptic function–related genes and altered expression of m^6^A modulators. Additionally, altered TAR DNA-binding protein 43 (TDP-43) function associated with m^6^A dysregulation may exacerbate HD pathology [57,58]. mHTT is associated with microglia-mediated inflammation and neuronal death [59]. Notably, abundant m^6^A modifications in mHTT transcripts suggest microglia-related pathogenic mechanisms linked to m^6^A RNA methylation [59,60,61] (Figure 2).

### 2.6. m^6^A Modifications of Microglia in MS

MS is an autoimmune neurodegenerative disease characterized by immune-mediated damage to the CNS, leading to inflammatory cell infiltration, demyelination, axonal injury, and reactive gliosis [62,63,64]. Cerebrospinal fluid from MS patients shows elevated expression of multiple m^6^A regulators (e.g., METTL3, METTL14, ALKBH5), though methylation levels are notably decreased in progressive MS compared to relapsing-remitting forms [65]. METTL3 deficiency in Th17 cells significantly reduces disease severity in experimental autoimmune encephalomyelitis (EAE), an animal model for MS, highlighting m^6^A’s role in disease progression [66].

Microglial activation contributes critically to MS pathogenesis through myelin debris clearance, cytokine release, and remyelination. Activation of TREM2 in microglia or antagonizing Gasdermin D (GSDMD)-mediated inflammation provides therapeutic potential for MS management. Notably, ALKBH5-mediated m^6^A modifications may influence microglial pyroptosis and inflammation through GSDMD, opening avenues for future MS research [67,68,69,70,71] (Figure 2).

### 2.7. m^6^A Modifications of Microglia in ALS

ALS involves extensive motor neuron degeneration, causing muscle atrophy, impaired mobility, and cognitive disturbances [72,73,74]. Studies report increased m^6^A methylation in ALS spinal cord tissue, with mutations in Fused in sarcoma (FUS) and TDP-43 linked to altered m^6^A signaling pathways contributing to disease pathology [75,76,77].

Microglial dysfunction significantly impacts ALS progression, transitioning from early neuroprotective roles to chronic neurotoxic and pro-inflammatory states [78,79,80,81]. C9orf72 gene repeat expansions lead to microglial activation deficits and increased ALS susceptibility [82]. Differential m^6^A modifications in immune-related genes, such as CX3CR1, further disrupt neuron–glia communication and exacerbate ALS pathology, highlighting the potential for future studies targeting microglial m^6^A regulation [83] (Figure 2).

Collectively, these findings underscore the importance of m^6^A RNA methylation in microglial function across inflammation in multiple neurodegenerative diseases, providing new insights and therapeutic targets for future research.

## 3. Astrocyte

### 3.1. Roles of Astrocytes in the CNS

Astrocytes fulfill critical roles in maintaining CNS homeostasis, supporting neuronal survival, regulating synaptic transmission, forming the blood–brain barrier (BBB), and facilitating tissue repair processes [84,85,86,87,88]. Although historically categorized into neurotoxic (pro-inflammatory A1) and neuroprotective (anti-inflammatory A2) phenotypes [89], emerging evidence indicates that astrocyte activation exhibits substantial heterogeneity beyond this binary classification, such as pro-inflammatory astrocytes differentiate into ACLY^+^EP300^+^ memory-like cells with an epigenetic phenotype, maintaining the persistent activation of inflammation-associated genes and enabling rapid and robust secondary inflammatory responses in MS [90], so necessitating deeper investigation into their diverse functions in neurological disorders.

### 3.2. m^6^A Modifications in Astrocyte

m^6^A RNA methylation is increasingly recognized as a critical regulator of astrocyte functions. For example, circHECW2-mediated inhibition of m^6^A methylation via downregulated WTAP contributes to astrocytic dysfunction in major depressive disorder (MDD) by decreasing guanine nucleotide binding-protein gamma subunit-4 (GNG4) mRNA levels [91]. ALKBH5 modulates glutamatergic transmission under chronic stress conditions, and its translocation blockade by circSTAG1 enhances FAAH mRNA degradation in astrocytes, reducing depressive behaviors in mice [92,93]. Additionally, ALKBH5-mediated suppression of LncRNA SNHG3 in astrocytes alleviates ischemic damage and inflammation induced by cerebral ischemia–reperfusion injury [94]. Furthermore, METTL3 enhances the stability of SOX2 mRNA, protecting glioma stem-like cells from radiation cytotoxicity [95], and METTL3-induced stabilization of nuclear paraspeckle assembly transcript 1 (NEAT1) and the activation of the miR-3773p/Nampt axis confer neuroprotection in ischemic injury [96]. METTL3 also mediates anti-inflammatory and anti-pyroptotic responses via NLRP3 downregulation in emodin-treated astrocytes [97]. Under neurotoxic stress, the epitranscriptomic reader YTHDF2 is responsible for the regulation of SEK1(MAP2K4)-JNK-c-JUN inflammatory signaling in astrocytes [98].

Moreover, m^6^A also facilitates astrocyte-associated tumorigenesis. It is reported that YTHDF2-mediated translation of circMET produces the MET404 variant, promoting glioblastoma tumorigenesis [99].

Reactive astrocytes play an important role in neurodegenerative diseases, contributing to both neuroinflammation and neuroprotection [100,101,102,103]. Alterations in m^6^A RNA modifications significantly influence these processes.

### 3.3. m^6^A Modifications of Astrocytes in AD

**Figure 3:**
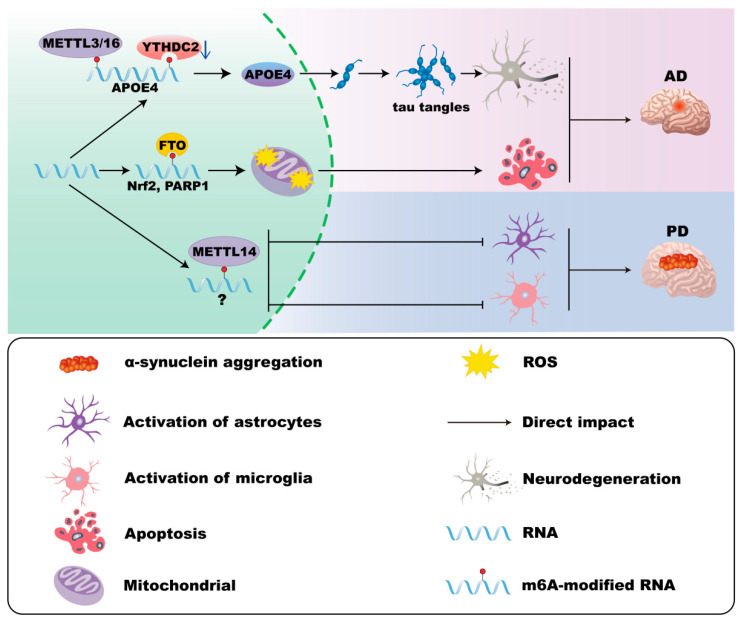
m^6^A modifications in astrocytes in AD and PD. In Alzheimer’s disease (AD), ApoE4 expression is associated with elevated METTL3/METTL16 and decreased YTHDC2 levels in astrocytes, which correlates with enhanced neurotoxicity. Moreover, FTO and YTHDF1 in astrocytes induce mitochondrial dysfunction and oxidative stress. In Parkinson’s disease (PD), the loss of Mettl14 in the substantia nigra increases astrocyte activation.

Astrocytic dysfunction in AD, including impaired calcium signaling, disrupted glutamate buffering, and enhanced synapse uptake via MFG-E8, exacerbates neuronal damage [104,105]. It has been found that ApoE4 expression correlates with enhanced neurotoxicity in AD, which is associated with elevated METTL3/METTL16 and decreased YTHDC2 levels [30,31,32,33,106]. Increased METTL3 and reduced ALKBH5 also promote astrocyte activation, intracellular aggregation of Microtubule-associated protein tau (MAPT), and neuroinflammation [107]. Moreover, elevated FTO and YTHDF1 in astrocytes induce mitochondrial dysfunction and oxidative stress, which can be mitigated by the FTO inhibitor MO-I-500 in AD [108] (Figure 3).

### 3.4. m^6^A Modifications of Astrocytes in PD

Astrocytes in PD demonstrate both protective roles, such as debris phagocytosis and inflammation inhibition [109,110], and detrimental roles, including pathogenic activation by α-synuclein and impaired protein clearance due to LRRK2 mutations [111,112]. Although direct evidence remains limited, loss of Mettl14 in the substantia nigra increases astrocyte activation, and manganese-induced downregulation of YTHDF2 exacerbates inflammatory responses, suggesting a potential role for m^6^A regulation in PD pathology [50,98] (Figure 3).

### 3.5. m^6^A Modifications of Astrocytes in HD

Although direct evidence linking m^6^A modifications in astrocytes to HD is absent, astrocytes significantly influence HD progression. They exhibit enhanced clearance of mutant huntingtin (mHTT), release protective cytokines like brain-derived neurotrophic factor (BDNF) and metallothionein-3 (MT3), but progressively lose normal functions due to mHTT aggregates [113,114,115,116,117,118,119,120]. Dysfunctional astrocytic cholesterol metabolism and impaired K+ buffering exacerbate neuronal vulnerability [121,122,123,124]. Gene expression changes, including reduced *µ-crystallin* (*Crym*), further reflect astrocytic dysfunction in HD [125].

### 3.6. m^6^A Modifications of Astrocytes in MS

In MS, astrocytes significantly contribute to neuroinflammation, demyelination, and impaired oligodendrocyte regeneration, exacerbating disease severity [126,127,128]. Current studies directly exploring astrocytic m^6^A in MS are scarce. Further support is found in spinal cord injury models, where METTL3-driven m^6^A modifications enhance astrocyte-mediated neuroprotection [129].

### 3.7. m^6^A Modifications of Astrocytes in ALS

Astrocytes in ALS exhibit significant molecular and functional heterogeneity, becoming toxic to motor neurons through mechanisms involving C9orf72-induced metabolic dysfunction, mutant SOD1 toxicity, and disrupted tripartite synapse plasticity [126,130,131,132]. Although no direct evidence currently links astrocytic m^6^A modifications to ALS pathology, mutations in RNA-binding proteins such as Fused in sarcoma (FUS) and TAR DNA-binding protein-43 (TDP-43), which are associated with altered m^6^A modifications, imply a potential epitranscriptomic mechanism that warrants further investigation [76,77,133,134]. Astrocytic m^6^A RNA modifications are crucial regulators of CNS homeostasis and pathology.

Notwithstanding the growing body of evidence, substantial knowledge gaps remain, especially regarding the direct roles of m^6^A modifications in astrocytes across various neurodegenerative diseases. Future investigations to clarify these epitranscriptomic mechanisms could potentially unveil novel therapeutic targets for CNS disorders.

## 4. Oligodendrocyte

### 4.1. Roles of Oligodendrocytes in CNS

Oligodendrocytes, derived from oligodendrocyte precursor cells (OPCs), are critical myelinating cells within the CNS [135]. Beyond myelination, they support axonal integrity and functionality [136], provide metabolic support [137], and modulate immune responses [138]. Dysfunctional oligodendrocytes are significantly implicated in various neurodegenerative diseases, including AD, PD, HD, MS, and ALS.

### 4.2. m^6^A Modifications in Oligodendrocyte

**Figure 4:**
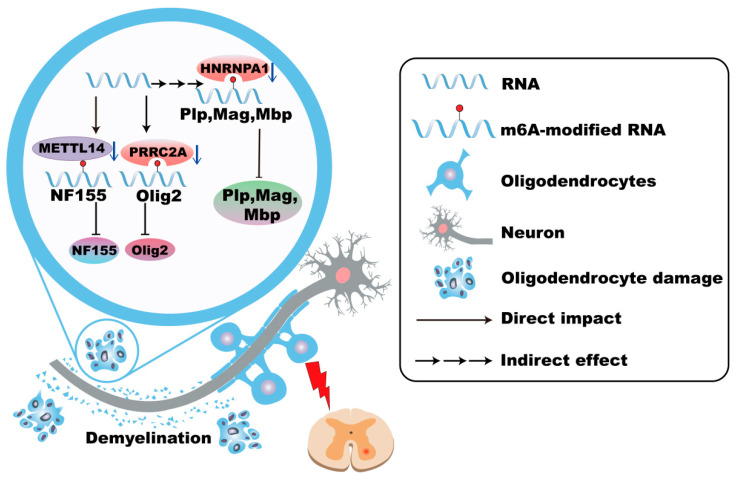
m^6^A modifications in oligodendrocytes in MS. In Multiple Sclerosis (MS), the absence of METTL14 in oligodendrocytes leads to abnormal splicing of transcripts such as NF155, disrupts paranodal junctions of myelin sheaths, and promotes demyelination. Genetic variations in PRRC2A are associated with increased MS susceptibility, which is related to the downregulation of Olig2. The dysfunction of hnRNP A1 leads to alterations in the expression of myelin-related genes and promotes the dysfunction and death of oligodendrocytes.

m^6^A RNA methylation is crucial for oligodendrocyte differentiation, maturation, and myelination processes. Mettl14 deficiency leads to a reduction in the number of mature oligodendrocytes and a decrease in myelin in the CNS [139]. In detail, loss of Mettl14 in oligodendrocytes causes aberrant splicing of transcripts such as neurofascin 155 (NF155), disrupting paranodal junctions and promoting demyelination [139,140]. Inflammatory cytokines, particularly IFN-γ, induce dysfunction in m^6^A reader heterogeneous nuclear ribonucleoprotein A1 (hnRNP A1), leading to altered expression of myelin-related genes (*Plp*, *Mag*, and *Mbp*) and promoting oligodendrocyte dysfunction and death [141]. Dysregulated m^6^A modifications in oligodendrocytes have been increasingly associated with neurodegenerative diseases, suggesting a potential therapeutic target (Figure 4).

### 4.3. m^6^A Modifications of Oligodendrocytes in AD

Oligodendrocytes significantly contribute to AD pathology involving myelin dysfunction, demyelination, and amyloid-beta (Aβ) peptide production [142,143]. Disease-associated oligodendrocytes, primarily located near Aβ plaques in AD patients, influence disease progression via ERK signaling [144]. Although direct evidence linking m^6^A modifications in oligodendrocytes and AD pathology remains limited, existing data suggest its important roles. Deletion of the m^6^A methyltransferase Mettl14 impairs oligodendrocyte differentiation, resulting in hypomyelination [139]. Furthermore, m^6^A readers such as Prrc2a stabilize Olig2 mRNA, a transcription factor critical for oligodendrocyte differentiation, and downregulation of Olig2 is linked to neuronal death in AD [145,146]. Additionally, Prrc2b deficiency destabilizes Sox2 mRNA, which is implicated in AD progression [147,148].

### 4.4. m^6^A Modifications of Oligodendrocytes in PD

Oligodendrocytes exhibit disease-specific molecular signatures in PD, characterized by inflammatory reprogramming and myelination abnormalities [149]. White matter abnormalities driven by myelin defects significantly contribute to PD pathology [150,151]. Oligodendrocytes prominently express leucine-rich repeat kinase 2 (LRRK2), a gene closely related to idiopathic and familial PD, highlighting their significance in PD pathogenesis [152,153,154]. Loss of oligodendrocyte-specific β-glucocerebrosidase triggers axonal degeneration, α-synuclein accumulation, astrogliosis, and lipid dyshomeostasis, exacerbating PD pathology [155]. Meanwhile, α-synuclein aggregates impair OPC differentiation and disrupt cellular energetics, possibly affecting age-related remyelination processes [156]. Although direct research into m^6^A modifications in oligodendrocytes in PD is limited, these pathways highlight oligodendrocytes as a potential target for future PD research.

### 4.5. m^6^A Modifications of Oligodendrocytes in HD

Oligodendrocyte dysfunction and associated white matter abnormalities constitute early pathological events in HD [55]. Mutant huntingtin protein enhances the activity of the polycomb repressive complex 2 (PRC2), causing delayed oligodendrocyte maturation and myelination deficits [157]. Metabolic disruptions involving glucose, thiamine, and lipid metabolism further impair oligodendrocyte maturation through pathways involving diacylglycerol (DAG) and protein kinase C epsilon (PRKCE). Dysfunction in thiamine metabolism, particularly thiamine pyrophosphokinase 1 (TPK1), is linked to behavioral deficits in HD mouse models [158]. Despite these findings, the direct role of m^6^A modifications in HD remains poorly understood, marking an important direction for future studies.

### 4.6. m^6^A Modifications of Oligodendrocytes in MS

Demyelination is a hallmark of MS, characterized by initial myelin sheath damage progressing toward oligodendrocyte cell body deterioration [159]. Oligodendrocyte pathology in MS is exacerbated by physiological aging and inflammation-induced cellular senescence [160]. Specific pathological events, including Gasdermin D (GSDMD) activation and Fas-mediated apoptosis, significantly contribute to oligodendrocyte damage and demyelination [70,161]. Genetic variations in m^6^A reader, PRRC2A, are associated with increased MS susceptibility, which is related to the downregulation of Olig2 [145,162]. Elevated levels of various m^6^A regulatory proteins (METTL3, METTL14, ALKBH5, FTO, WTAP, etc.) are observed in cerebrospinal fluid from MS patients, suggesting their potential roles in MS pathogenesis [65] (Figure 4).

### 4.7. m^6^A Modifications of Oligodendrocytes in ALS

ALS pathology involves motor neuron degeneration accompanied by oligodendrocyte dysfunction and impaired myelination [163]. Reduced numbers of m^6^A-positive oligodendrocytes in ALS patient brains suggest that impaired m^6^A methylation may contribute to disease pathology [164]. Additionally, the RNA-binding protein TDP-43, crucial for oligodendrocyte survival and myelin integrity, is regulated by m^6^A modifications. Loss of TDP-43 leads to progressive myelin degeneration, highlighting a key connection between m^6^A modification, TDP-43 dysfunction, and ALS pathogenesis [75,165]. This interaction presents a promising target for novel therapeutic strategies in ALS.

## 5. Neuron

### 5.1. Roles of Neurons in CNS

Neurons serve as the fundamental units of the nervous system. They establish intricate networks via synaptic connections, which are essential for mediating perception, cognition, motor function, and memory processes [166,167]. Proper neuronal function is vital for the overall health and operation of the nervous system.

### 5.2. m^6^A Modifications in Neuron

m^6^A RNA modification is crucially involved in neuronal health, influencing learning, memory, aging, and neurodegeneration. Notably, neuronal m^6^A-modified transcripts exhibit lower translational efficiency compared to glial cells, underscoring distinct roles of m^6^A across cell types [168,169]. This section reviews recent insights into neuronal m^6^A modifications in neurodegenerative diseases.

### 5.3. m^6^A Modifications of Neurons in AD

**Figure 5:**
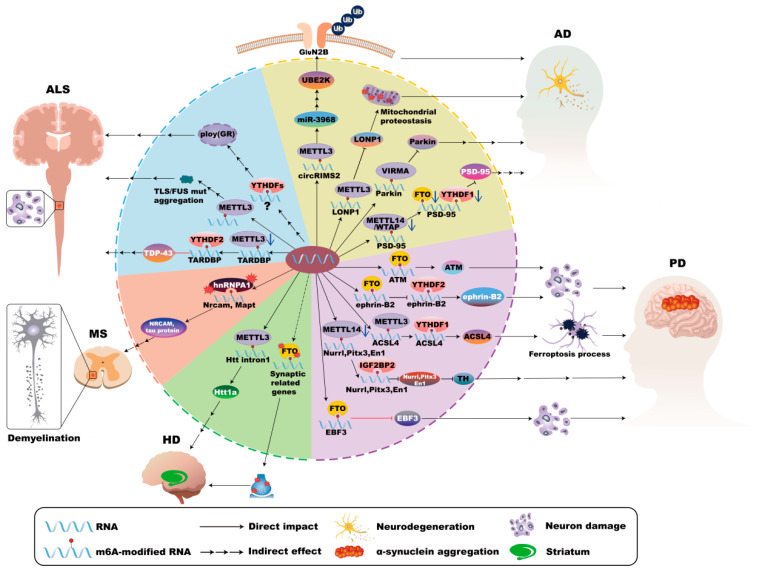
m^6^A modifications in neurons in neurodegenerative disease. In Alzheimer’s disease (AD), METTL3-mediated m^6^A modification activates the circRIMS2/miR-3968 pathway, causing abnormal activation of UBE2K, subsequent degradation of GluN2B, and synaptic impairment. Dysregulation of METTL3 disrupts mitochondrial proteostasis via the LONP1 complex. Moreover, increased VIRMA activity leads to heightened m^6^A modification of PRKN RNA, reducing its stability and consequently impairing mitophagy and neuronal death. METTL13 deficiency diminishes PSD95 expression, contributing to synaptic defects. In Parkinson’s disease (PD), FTO expression reduces m^6^A levels, stabilizing ATM mRNA and consequently promoting dopaminergic neuron death. Manganese exposure disrupts neuronal projections through the FTO/ephrin-B2/YTHDF2 pathway, which underscores the sensitivity of neuronal m^6^A machinery to external insults. Furthermore, Soot nanoparticles increase METTL3 and YTHDF1 expression, amplifying ACSL4-mediated ferroptosis. However, METTL14 deficiency reduces the expression of essential transcription factors (Nurr1, Pitx3, En1) and impacts the Tyrosine hydroxylase (TH) expression and dopaminergic functions. Increased m^6^A methylation of Early B-cell factor 3 (EBF3) mRNA stabilizes its expression, ameliorating motor deficits and inhibiting apoptosis in PD. In Huntington’s Disease (HD), METTL3-mediated m^6^A methylation of the Huntingtin gene (Htt1a) correlates with HD progression. Impairment of cognitive-training-induced changes in FTO distribution disrupts synaptic gene expression and memory functions. In Multiple Sclerosis (MS), dysfunctional hnRNP A1 disrupts RNA splicing of critical neuronal genes (*Mapt* and *Nrcam*), resulting in functional damage of neurons. In Amyotrophic lateral sclerosis (ALS), the loss of METTL3 activity elevates TDP-43 expression by reducing m^6^A modification of TARDBP, disrupting neuronal homeostasis. However, Mutations in the RNA-binding protein FUS are associated with elevated neuronal m^6^A levels. m^6^A-modified RNAs and YTHDF proteins promote poly (GR) inclusions, intensifying neuronal toxicity and disease progression.

In AD, neuronal dysfunction manifests through amyloid plaques, neurofibrillary tangles, synaptic loss, and neuronal death, causing cognitive decline [170,171,172,173,174]. Several studies have elucidated the significant roles of m^6^A modifications in AD pathogenesis. METTL3-mediated m^6^A modification activates the circRIMS2/miR-3968 signal, resulting in aberrant Ubiquitin-conjugating enzyme E2K (UBE2K) activation, subsequent GluN2B degradation, and synaptic impairment associated with memory deficits [175]. Additionally, dysregulation of METTL3 induced by Aβ42 disrupts mitochondrial proteostasis via the Lon protease homolog 1 (LONP1) complex, exacerbating mitochondrial dysfunction [176]. METTL13 deficiency has also been reported to diminish postsynaptic density protein 95 (PSD95) expression, further contributing to synaptic defects and neurodegeneration [27]. However, METTL3 mediates the m^6^A modification of Leucine-rich repeat and immunoglobulin containing nogo receptor 2 (Lingo2), which promotes the degradation of Lingo2 mRNA and facilitates the production of Aβ in AD mice [177]. Furthermore, increased VIRMA activity leads to heightened m^6^A modification of PRKN mRNA, reducing its stability and consequently impairing mitophagy, which promotes neuronal death [3]. Moreover, exposure to 27-Hydroxycholesterol (27-OHC) decreases m^6^A methylation levels by downregulating key regulators (METTL14, YTHDF1, FTO), thereby reducing synapse-associated molecules and impairing cognition. Interestingly, the intestinal commensal bacterium Roseburia intestinalis can reverse these detrimental effects, suggesting potential therapeutic approaches [178] (Figure 5).

### 5.4. m^6^A Modifications of Neurons in PD

PD is characterized by the degeneration of dopaminergic neurons in the substantia nigra, which is closely associated with altered neuronal m^6^A modifications. Elevated FTO expression reduces m^6^A levels, stabilizes Ataxia telangiectasia mutated (ATM) mRNA, and consequently promotes dopaminergic neuron death [179]. However, in another study, Salsolinol, a catechol isoquinoline that causes neurotoxicity, increases the level of m^6^A modification by inhibiting the expression of FTO and ALKBH5 in neurons, which led to the downregulation of Yes-associated protein 1 (YAP1), promoting autophagy and the death of dopaminergic neurons [180]. Environmental factors such as manganese exposure disrupt neuronal projections through the Foxo3a/FTO/ephrin-B2/YTHDF2 signaling, which underscores the sensitivity of neuronal m^6^A machinery to external stress [181]. Additionally, soot nanoparticles have been shown to increase METTL3 and YTHDF1 expression, amplifying ACSL4-mediated ferroptosis and exacerbating neuronal loss in PD [15]. FTO mediates the m^6^A demethylation of BRCA1-associated protein 1 (BAP1) and promotes its upregulation, resulting in the death of dopaminergic neurons by inducing ferroptosis [182]. Therapeutically, FTO inhibitors exhibit promising neuroprotective effects in PD models [183]. Furthermore, Mettl14-mediated m^6^A modification acts on transcription factors Nurr1, Pitx3, and EN1, thereby regulating the expression of tyrosine hydroxylase (TH) and its related functions. METTL14 deficiency reduces the expression of these essential transcription factors and impairs dopaminergic neuron function [50,184,185]. METTL14 binds to and modifies the m^6^A motif in the coding region of α-synuclein (α-syn) mRNA, while the reader protein YTHDF2 participates in recognizing m^6^A-modified α-syn mRNA and impairs its stability. However, the mRNA levels of m^6^A, METTL3, METTL14, and YTHDF2 are significantly reduced in PD patients, which may be associated with the pathogenesis of PD [47]. Conversely, NRF1-mediated upregulation of METTL3 elevates glutaredoxin (GLRX) expression, supporting neuronal survival through enhanced RNA stability regulated by IGF2BP2 [186,187]. Similarly, increased m^6^A methylation of EBF3 mRNA stabilizes its expression, ameliorating motor deficits and inhibiting apoptosis, thus providing symptomatic relief in PD [188] (Figure 5).

### 5.5. m^6^A Modifications of Neurons in HD

HD primarily refers to medium-sized spiny neurons in the striatum [189], and altered m^6^A modifications contribute significantly to its pathology. Research using Hdh+/Q111 mouse models of HD demonstrates that impaired cognitive-training-induced alterations in nuclear METTL14 and FTO distribution disrupts synaptic gene expression and memory functions. Pharmacological inhibition of m^6^A demethylation ameliorates these cognitive deficits [57]. Additionally, increased m^6^A methylation of the Huntingtin gene (*Htt1a*) correlates with HD progression, which is influenced by METTL3 enzymatic activity [61]. Furthermore, TDP-43 dysfunction in conjunction with altered m^6^A modification patterns affects RNA splicing and gene regulation, contributing to neuronal dysregulation and the pathogenic expansion of CAG repeats characteristic of HD [58,190] (Figure 5).

### 5.6. m^6^A Modifications of Neurons in MS

MS involves inflammation-driven neuronal injury, where alterations in m^6^A modifications significantly impact disease progression. Dysfunctional hnRNP A1, an important m^6^A reader, disrupts RNA splicing of critical neuronal genes, including *Mapt* and *Nrcam*, resulting in neurite damage and impaired neuronal functionality [191,192]. Environmental aluminum exposure further exacerbates neuronal injury by downregulating key m^6^A regulators (METTL3, METTL14, FTO, and YTHDF2), causing global reductions in m^6^A RNA methylation and subsequent neuronal dysfunction [193,194,195] (Figure 5).

### 5.7. m^6^A Modifications of Neurons in ALS

ALS, characterized by motor neuron degeneration, is closely linked to dysregulated neuronal m^6^A modifications. The loss of METTL3 activity in cholinergic neurons elevates TDP-43 expression by reducing m^6^A modification of TARDBP, then disrupts neuronal homeostasis [77]. YTHDF2 is also implicated in mediating TDP-43 toxicity, and knockout of YTHDF2 alleviates neurodegeneration [75]. Additionally, mutations in the RNA-binding protein FUS are associated with elevated neuronal m^6^A levels, suggesting pathogenic interactions that can be mitigated by inhibiting METTL3 in ALS [76,133]. ALS is associated with *C9orf72* gene repeat expansions, which involve downregulated METTL3 and METTL14 expression and result in global m^6^A hypomethylation. This disrupts RNA metabolism and impairs neuronal function, primarily through dysregulated glutamate synapses and calcium signaling pathways [196,197]. Furthermore, YTHDFs promote poly(GR) inclusion formation and intensify neuronal toxicity and disease progression [198]. Moreover, downregulation of the m^6^A reader RNA binding motif protein X-linked (RBMX) can induce the activation of the p53 pathway, resulting in neuronal defects and the progression of ALS [199] (Figure 5).

Neuronal m^6^A modifications significantly influence neurodegenerative diseases by modulating essential biological processes, including RNA metabolism, synaptic integrity, mitochondrial homeostasis, and neuronal survival. Continued investigation into these pathways could reveal novel therapeutic strategies for neurodegenerative disorders.

## 6. Conclusions and Perspectives

Research on neurodegenerative diseases currently spans multiple domains, focusing on the misfolding and aggregation of proteins such as Tau, α-synuclein, and TDP-43, along with neurotoxic effects, as well as neuroinflammation involving glial cells. Recently, RNA modifications, specifically m^6^A, have emerged as critical regulators in glial cells and neurons in neurodegenerative diseases (Table 1 and Table 2).

However, research into m^6^A modifications in neurodegenerative diseases is still in the early stages, and most studies focus primarily on AD and PD. Investigations into other neurodegenerative diseases remain exploratory. Neuronal populations have been the primary focus of research on m^6^A modification dynamics, while studies of glial cells remain limited despite their critical roles in neurodegeneration.

In AD, research on m^6^A modifications has advanced significantly, spanning both neurons and glial cells populations. By contrast, in MS, initial studies focusing on oligodendrocytes have emerged, but substantial knowledge gaps remain for other glial cell types. A further critical limitation is the lack of specificity in m^6^A modification site mapping, and precise cellular localization in CNS. Given the distinct biological functions of neurons and glial cells, future research must prioritize the detailed identification of cell-specific m^6^A sites.

Additionally, current studies have demonstrated alterations in m^6^A modification in individual neurodegenerative diseases, but fail to identify the initiating factors driving this cascade of changes. In this review, we summarize m^6^A modifications from the perspective of distinct cells. It is evident that further investigations are needed to elucidate how m^6^A modifications mediate crosstalk between different cell populations.

Furthermore, the mapping of cell-type-specific m^6^A landscapes in the brain, the crosstalk between m^6^A and other epigenetic modifications (e.g., 5-methylcytosine [m5C] and histone lactylation), as well as the temporal dynamics of m^6^A modifications, remain to be fully elucidated.

Currently, the discovery of various m^6^A modification-related protein modulators has led to the development of chemical agents that show therapeutic promise, particularly in cancer. Nevertheless, the role of m^6^A in neurodegenerative diseases requires further elucidation. Apelin-13, a neuropeptide, upregulates the expression of METTL3 through an m^6^A-dependent mechanism, so it downregulates the lncRNA BDNF-AS, thereby suppressing neuroinflammation and activating the BDNF/TrkB pathway to ameliorate Alzheimer’s disease in rats [200]. Therapeutic strategies targeting m^6^A modification have opened up a new potential direction for the treatment of neurodegenerative diseases. By regulating RNA methylation status to interfere with disease-related molecular pathways, such as leveraging CRISPR-dCas13 to target and recruit m^6^A regulators to specific RNA loci of disease-related genes. These strategies have demonstrated promising application prospects in alleviating neuroinflammation, protecting neuronal survival, and improving synaptic function. However, these strategies currently face key challenges, including insufficient selectivity and off-target effects, which may impair therapeutic efficacy and pose potential safety risks. Therefore, future research needs to closely integrate cell-type-specific m^6^A mechanisms to further optimize therapeutic tools. For example, developing cell-type-targeted delivery systems for m^6^A modulators to enable their precise action on specific cells in the CNS. This will avoid damage to other tissues and cells, thereby reducing side effects in clinical practice.

In summary, research on m^6^A RNA modification holds significant promise for uncovering novel mechanisms and therapeutic strategies in neurodegenerative diseases. Despite existing limitations, including the need for enhanced cellular localization specificity and broader disease coverage, advancing our understanding of m^6^A modifications could substantially improve therapeutic outcomes for neurodegenerative diseases.

## Figures and Tables

**Table 1:** The role of m^6^A modification in glial cells in neurodegenerative diseases.

Cell	Diseases	m^6^A Regulators	Downstream Pathways	References
Microglia	AD	METTL3, METTL16, YTHDC2	APOE4 and tau-related neurodegeneration and lipid peroxidation	[30,31,32,33]
		YTHDF2	NLRP3/caspase-1/GSDMD pathway	[34]
		METTL3/ IGF2BP2	microglia M1/M2 polarization	[35]
	PD	VIRMA	Decreased Parkin, increased microglial activation, and dopaminergic neuron loss	[3,48,49]
		METTL14	Enhanced microglial activation	[50,51]
	ALS	METTL3, METTL14	Disorder of CX3CR1 signaling, disruption of neuron–glia communication	[83]
Astrocyte	AD	METTL3, YTHDC2, METTL16	Activation of astrocytes and neurotoxicity	[30,31,32,33,106]
		METTL3, ALKBH5	MAPT pathology, astrocyte activation	[107]
		FTO, YTHDF1	Oxidative stress and apoptosis, mitochondrial dysfunction, and energy metabolism disorders	[108]
	PD	METTL14	Enhanced astrocyte activation	[50]
Oligodend-rocytes	MS	METTL14	The decrease in NF155	[139,140]
		hnRNP A1	Altered expression of *Plp*, *Mag*, and *Mbp*	[141]
		PRRC2A	Downregulation of Olig2	[145,162]

AD: Alzheimer’s disease; ALKBH5: AlkB homolog H5; ALS: Amyotrophic lateral sclerosis; APOE4: Apolipoprotein E4; FTO: fat mass and obesity-associated protein; GSDMD: Gasdermin D; HD: Huntington’s disease; hnRNP A1: heterogeneous nuclear ribonucleoprotein A1; IGFBPs: Insulin-like growth factor-binding protein 2; MAPT: Microtubule-associated protein tau; METTL3/14/16: methyltransferase-like enzyme 3/14/16; MS: Multiple Sclerosis; NLRP3: NLR family pyrin domain containing 3; Olig2: Oligodendrocyte transcription factor 2; PD: Parkinson’s disease; PRRC2A: Proline-rich Coiled-coil 2a; VIRMA: Vir-like N6-methyladenosine (m^6^A) methyltransferase-associated protein; YTHDF1/2/3: YTH structural domain family protein 1/2/3.

**Table 2:** The role of m^6^A modification in neurons in neurodegenerative diseases.

Diseases	m^6^A Regulators	Downstream Pathways	References
AD	METTL3	The upregulates circRIMS2/miR-3968 pathway and GluN2B degradation	[175]
		Impaired LONP1 Complex and damage mitochondrial proteostasis and function	[176]
		Diminished PSD95 expression	[27]
		Degradation of Lingo2 mRNA and Aβ production	[177]
	VIRMA	Reduce the stability of PRKN RNA, mitophagy	[3]
	METTL4, YTHDF1, FTO	Reduced synapse-associated molecules	[178]
PD	FTO	Stabilization of ATM mRNA and dopaminergic neuron death	[179]
	FTO, ALKBH5	downregulation of YAP1	[180]
	FTO, YTHDF2	Degradation of ephrin-B2 mRNA	[181]
	METTL3, YTHDF1	ACSL4-mediated ferroptosis	[15]
	FTO	Upregulation of BAP1	[182]
	METTL14	Reduces the expression of Nurr1, Pitx3 and En1	[50,184,185]
	METTL3, IGF2BP2	Reduced m^6^A methylation level of GLRX	[186,187]
	FTO, METTL14, METTL3	Downregulate the expression of EBF3	[188]
HD	METTL14, FTO	Disrupted the translation or proper processing of synaptic genes	[57]
	METTL3	The expression of Htt1a	[61]
MS	hnRNP A1	altered neuronal RNA splicing of *Mapt* and *Nrcam*	[191,192]
	METTL3, METTL14, FTO, YTHDF2	Al-induced neurotoxicity	[193,194,195]
ALS	METTL3	upregulation of TDP-43	[77]
	YTHDF2	TDP-43 related toxicity	[75]
	METTL3	The mutation of FUS	[76,133]
	METTL3, METTL14	dysregulation of RNA metabolism, dysregulated glutamate synapses and calcium signaling	[196,197]
	YTHDF1, YTHDF3	promote poly (GR) inclusion formation	[198]
	RBMX	Activation of the p53 pathway	[199]

ACSL4: Acyl-CoA synthetase long-chain family member 4; AD: Alzheimer’s disease; ALKBH5: AlkB homolog H5; ALS: Amyotrophic lateral sclerosis; ATM: Ataxia telangiectasia mutated; BAP1: BRCA1-associated protein 1; EBF3: Early B-cell factor 3; FTO: fat mass and obesity-associated protein; FUS: Fused in sarcoma; HD: Huntington’s disease; hnRNP A1: heterogeneous nuclear ribonucleoprotein A1; IGFBPs: Insulin-like growth factor-binding protein 2; Lingo2: Leucine-rich repeat and immunoglobulin containing nogo receptor 2; LONP1: Lon protease 1; METTL3/14/16: methyltransferase-like enzyme 3/14/16; MS: Multiple Sclerosis; PD: Parkinson’s disease; PRRC2A: Proline-rich Coiled-coil 2a; PSD-95: Postsynaptic density protein-95; TDP-43: TAR DNA-binding protein-43; VIRMA: Vir-like N6-methyladenosine methyltransferase-associated protein; WTAP: WT1-associated protein; YAP1: Yes-associated protein 1; YTHDF1/2/3: YTH structural domain family protein 1/2/3.

## Data Availability

Not applicable.

## Abbreviations

ACSL4	Acyl-CoA synthetase long-chain family member 4
AD	Alzheimer’s disease
ALKBH5	AlkB homolog H5
ALS	Amyotrophic lateral sclerosis
APOE4	Apolipoprotein E4
ATM	Ataxia telangiectasia mutated
BAP1	BRCA1-associated protein 1
EBF3	Early B-cell factor 3
En1	Engrailed 1
FTO	Fat mass and obesity-associated protein
FUS	Fused in sarcoma
GLRX	Glutaredoxin
HD	Huntington’s disease
hnRNP A1	Heterogeneous nuclear ribonucleoprotein A1
Htt intron1	Huntingtin gene
IGF2BP2	Insulin-like growth factor 2 mRNA-binding protein 2
KDM3A	Lysine demethylase 3A
Lingo2	Leucine-rich repeat and immunoglobulin-containing
LONP1	Lon protease 1
Mag	Myelin-associated glycoprotein
MAPT	Microtubule-associated protein tau
Mbp	Myelin basic protein
METTL3/14/16	Methyltransferase-like enzyme 3/14/16
MS	Multiple Sclerosis
NF155	Neurofascin 155
NLRP3	NLR family pyrin domain containing 3
NRCAM	Neuronal cell adhesion molecule
Nrf2	NF-E2-related factor 2
Olig2	Oligodendrocyte transcription factor 2
PARP1	Poly (ADP-ribose) polymerase 1
PD	Parkinson’s disease
Pitx3	Paired-like homeodomain transcription factor 3
*Plp*	Proteolipid protein
PRRC2A	Proline-rich Coiled-coil 2a
PSD-95	Postsynaptic density protein-95
RBMX	RNA-binding motif protein X-linked
Salsolinol	1-methyl-6,7-dihydroxy-1,2,3,4-tetrahydroisoquinoline
TARDBP	Transactive Response DNA Binding Protein
TDP-43	TAR DNA-binding protein-43
TH	Tyrosine hydroxylase
UBE2K	Ubiquitin-conjugating enzyme E2K
VIRMA	Vir-like N6-methyladenosine Methyltransferase-associated protein
WTAP	WT1-associated protein
YAP1	Yes-associated protein 1
YTHDF1/2/3	YTH structural domain family protein 1/2/3
